# The sound stimulation method and EEG change analysis for development of digital therapeutics that can stimulate the nervous system: Cortical activation and drug substitution potential

**DOI:** 10.1111/cns.14014

**Published:** 2022-11-15

**Authors:** Deachang Kim, JaeHyun Woo, Jeahoon Jeong, Sungmin Kim

**Affiliations:** ^1^ Department of Medical Biotechnology Dongguk University‐Bio Medi Campus South Korea; ^2^ Department of R&D Support Research Institute for Commercialization of Biomedical Convergence Technology Seoul South Korea; ^3^ Research Institute for Commercialization of Biomedical Convergence Technology Dongguk University South Korea

**Keywords:** digital therapeutics, drug replacement, electroencephalogram, sound stimulation

## Abstract

**Introduction:**

The purpose of this study is to propose a treatment method and the effect on the nervous system of digital therapeutics, which is a new treatment method to replace surgery and drug prescription for the treatment and prevention of diseases.

**Methods:**

The 20 subjects who participated in the experiment, including men and women, had an average age of 26 ± 2.40 years. The proposed treatment method used three types of sound stimulation and air or bone conduction sound transmission methods to induce total of 6‐time EEG electroencephalogram(EEG) changes. EEG was measured with 200 sampling rate each in the P4, Cz, F8 and T7 channel located in the parietal, central, frontal and temporal lobes, respectively, according to the 10/10 system. A total of 2 min of data were created by extracting EEG signals with less noise from the measured data and the extracted data were applied with a 1–40 Hz Butterworth filter and a 50 Hz notch filter with a quality factor of 30. After that, EEG are subdivided into delta (0.5–4 Hz), theta (4–8 Hz), alpha (8–13 Hz), beta (13–30 Hz), and gamma (30–45 Hz) bands. Finally, EEG changes in response to sound stimuli were analyzed using power spectral density and T‐test validation in the frequency band.

**Results:**

When a sound stimulus of less than 1 KHz was stimulated by air conduction, brainstem activation was induced and the reticular activation system was activated. In addition, a great potential for replacing drugs was confirmed by inducing changes in the nervous system similar to drugs used for sedation.

**Conclusion:**

These results will be able to expand the concept of digital therapeutics, and it is expected that it will be developed as a safer treatment method that can replace surgery and drugs.

## Graphical abstract

Digital therapeutics using sound stimulation has the potential to be used as a safe treatment method that can replace neuropharmacological treatment and electrical stimulation. Stimulation of the auditory nerve using neutral music and air conduction can induce the activation of the reticular activating system. Finally, It was confirmed that the stimulation has a value that can replace drugs by inducing changes in the nervous system similar to those induced by propofol for the sedative effect.

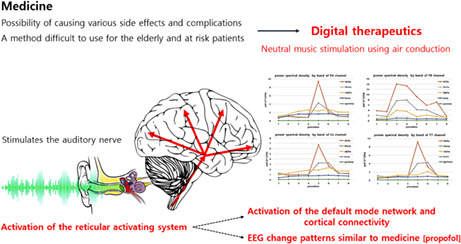

## 1 INTRODUCTION

The nerves in the human body largely compose the central nervous system (CNS), which is in charge of the brain and spinal cord, and the peripheral nervous system (PNS), which is in charge of nerves other than the brain and spinal cord. Among them, the PNS is composed of the somatic nervous system, which is controlled by the cerebrum, and the autonomic nervous system, which is not controlled by the cerebrum, and it controls and regulates other organs in the body.^1^ This process represents the antagonistic action of maintaining homeostasis in the body.

The CNS is connected to almost all parts of the body with the brain as the main axis, and the brain is the center of the nervous system. Basically, the brain processes and interprets the information and gives instructions based on the results. The PNS uses afferent and efferent nerves to provide a feedback to the CNS in real time.^2^ Afferent neurons sense external stimulation and transmit sensory information to the CNS, and efferent neurons transmit motor information from the CNS to body organs.

This feedback action results in synchronized synaptic activity in a population of cortical neurons. After a synaptic action, it creates a relative voltage difference between neurons.^3^ Thus, the electrical activity generated by the biochemical interaction of brain neurons develops, and its noninvasive measurement is called electroencephalography (EEG). EEG is used as a primary method for diagnosing tumors, stroke, and brain disorders.^4^, ^5^, ^6^, ^7^, ^8^ However, it is very difficult to observe the brain nervous system using EEG in the time domain because it is a waveform that vibrates in a very complex pattern. For this reason, cross wavelet correlation and power spectral density (PSD), which classify and observed frequency domain, are mainly used when observing EEG. This analysis method divides the subbands of EEG into delta, theta, alpha, beta, and gamma bands and assigns power units to each frequency unit to improve periodicity, so that meaningful results can be derived from signals with complex patterns.^9^, ^10^

The study and development of EEG has become an effective measurement method and indicator that can confirm the reversible and progressive causes of the nervous system disorders caused by aging, infection, and vascular problems. Accordingly, various studies are being performed to identify the brain and nervous system that change according to various methods and effects for treating diseases.^11^, ^12^, ^13^, ^14^, ^15^ However, among various treatment methods, adverse events of EEG and the risk and side effects of epileptic seizures were confirmed in neuropharmacological therapy using antipsychotic medication.^16^, ^17^

In order to solve these side effects, research on brain disease treatment is gradually changing from neuropharmacological therapy to research using noninvasive stimulation.^18^ Representative techniques are transcranial electrical stimulation and noninvasive nerve stimulation. Electrical stimulation has emerged as an attractive treatment option for treating the cranial nerves for abnormal cognitive, motor functions and the abnormal autonomic nervous system.^19^, ^20^, ^21^ However, although noninvasive stimulation is used, a safer neurological treatment method is being studied according to the negative probability that it may cause various side effects and complications.^22^

Accordingly, in this study, sound stimulation using a noninvasive method and the effect of a new treatment technique were analyzed and confirmed by EEG analysis of the nervous system that changes according to the stimulation method. One of these treatments, the digital therapeutics, is based on a software program to prevent and treat patients' diseases and disorders.^23^ Sound can be digitized by using sample rate, frequency, and recording, and since various changes are possible, it was judged that there is a possibility as a digital therapeutics using software and we want to check the possibility.

## 2 METHODS

### 2.1 The hypothesis of nerve stimulation

The cranial nerves originate from the brain and are generally classified into 12 pairs (Table 1). These 12 pairs of cranial nerves are connected to the reticular formation, a structure of nerve fiber bundles that exist continuously in the brainstem centers of the diencephalon, midbrain, pons, and medulla. The reticular formation is involved in the cerebral cortical activity that covers all areas of the brain. This is called the reticular activating system (RAS).^24^

**TABLE 1:** Types and components of the 12 pairs of cranial nerves

Number	Name	Type	Components	Area
1	Olfactory nerve	Sensory	SVA	Telencephalon
2	Optic nerve	Sensory	SSA	Diencephalon
3	Oculomotor nerve	Motor	GSE, GVE	Midbrain
4	Trochlear nerve	Motor	GSE	Hindbrain
5	Trigeminal nerve	Mixed	GSA, GVA, SVE	Pons
6	Abducens nerve	Motor	GSE	
7	Facial nerve	Mixed	GSA, GVA, GVE, SVA SVE	
8	Acoustic nerve	Sensory	SSA	
9	Glossopharyngeal nerve	Mixed	GSA GVA GVE SVA SVE	Medulla
10	Vagus nerve	Mixed	GSA GVA GVE SVA SVE	
11	Accessory nerve	Motor	GSE SVE	
12	Hypoglossal nerve	Motor	GSE	

Abbreviations: GSA, general somatic afferent; GSE, general somatic efferent; GVA, general visceral afferent; GVE, general visceral efferent; SSA, special somatic afferent; SVA, special visceral afferent; SVE, special visceral efferent.

**FIGURE 1:**
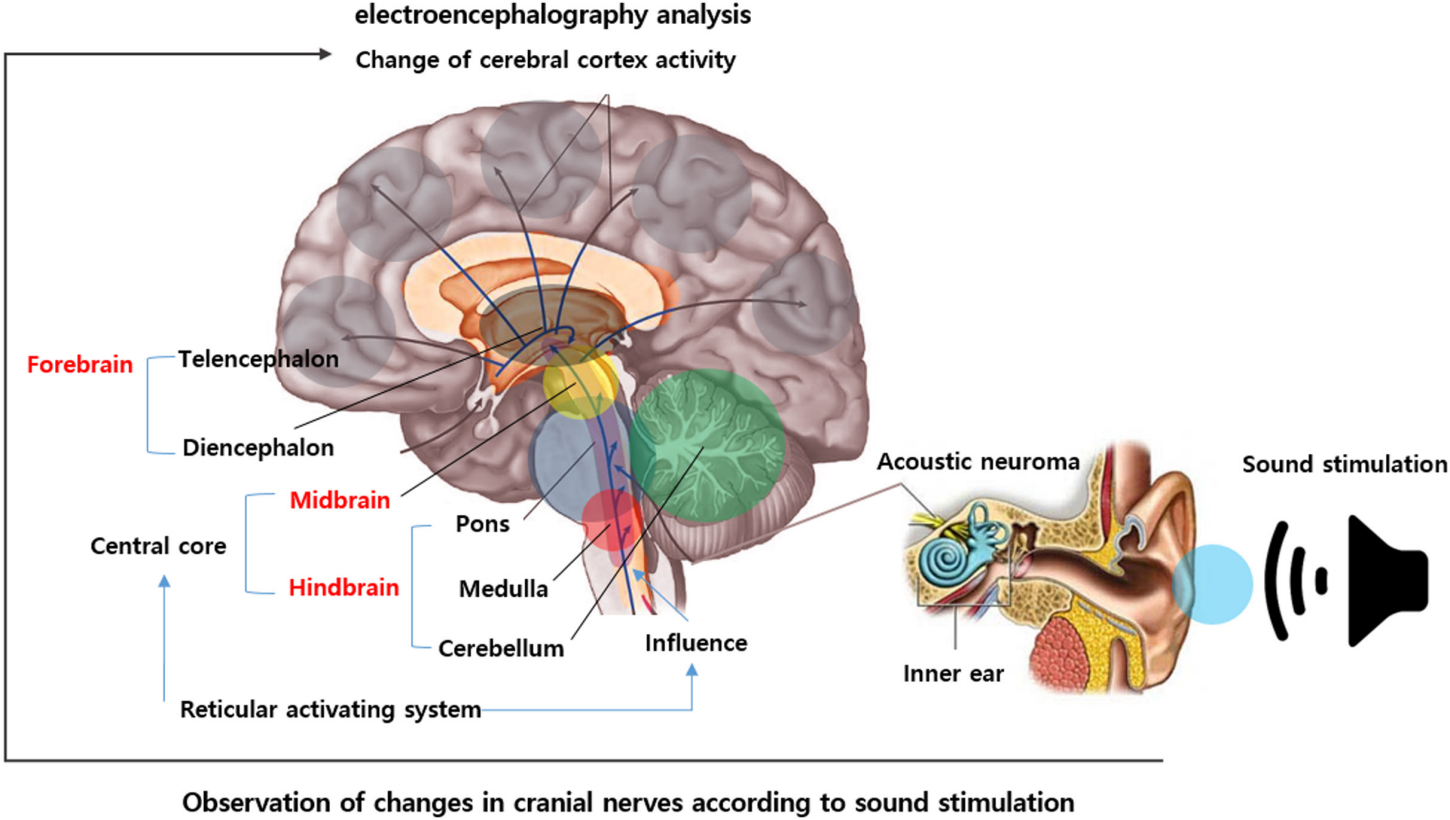
Sound stimulation research hypothesis for stimulating the reticular activation system. Sound stimulation will induce brain activity as a new therapeutic alternative to drugs and electrical stimulation. We hypothesized that auditory nerve stimulation could stimulate the reticular activation system. The reason is that the primary location of the reticular activation system is that the pons contained in the brainstem receive information from the auditory nerve

In this study, among the sensory types included in the RAS, we expected that the reticulum activation system would be activated when the acoustic nerve was selectively stimulated^25^ and that changes in cortical activity would be observed on the EEG (Figure 1). In addition, since the pons, to which the acoustic nerve is connected, performs information transmission between the cerebellum and the cerebrum, it was expected to cause various changes in the body according to the stimulation.

### 2.2 The sound stimulation method and data measurement

**FIGURE 2:**
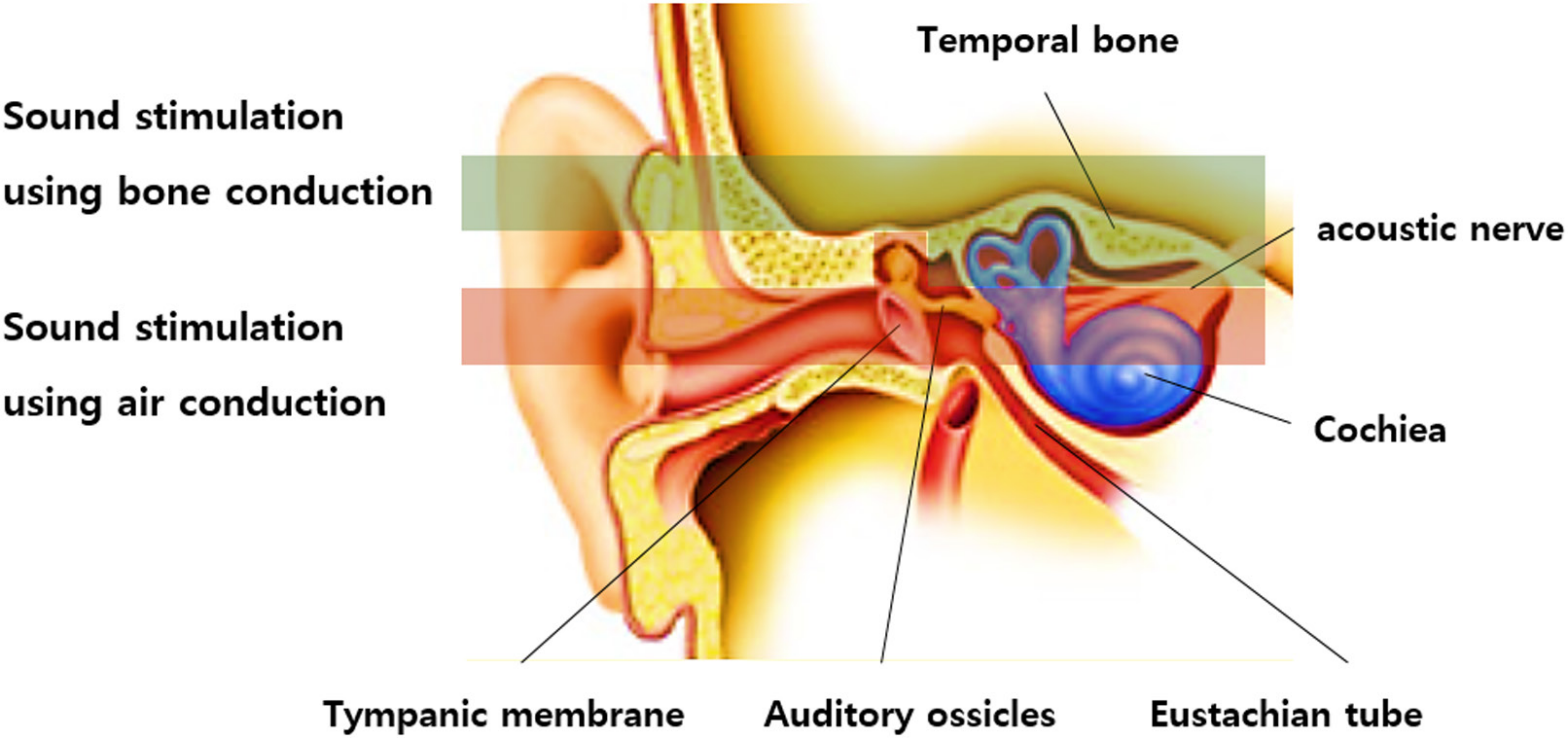
Sound stimulation delivery method. In the study, air conduction, which is generally used, and bone conduction, which is transmitted through the vibration of the skull, were used. The biggest reason for using both methods is the organ of Corti in the cochlea. The inner part of the organ of Corti stimulates the auditory nerve in the low frequency band. However, the purpose of this study was to determine the possibility of energy transfer sufficient to change the acoustic nerve stimulation and brain waves in the low frequency band in vibration using bone

Methods for stimulating the acoustic nerve are divided into air conduction and bone conduction. In air conduction, sound waves enter the ear, pass through the eardrum and ossicles, and stimulate the inner ear. Bone conduction produces vibrations of the skull, stimulating the inner ear directly (Figure 2). These stimulations produce vibrations of the lymph fluid in the cochlea, and the acoustic nerve within the vibration frequency band responds and transmits signals. Both methods can lead to the same, similar sound potential through research.^26^, ^27^

Accordingly, the auditory evoked potential EEG‐Biometric dataset^28^ was used in this study. This open data set included resting state and auditory stimulation experiments of 20 subjects (Table 2). The subjects were recruited by male and female, and had an average age of 26 ± 2.40 and an average body mass index of 24.89 ± 4.44. Subjects were asked to sit and rest in a comfortable chair, and the recordings were performed for 3 min each according to the procedure in Table 2. In the subject's auditory stimulation experiment, the change in sound stimulation was a total of six times. Native music, foreign language music, and natural music were used as sound stimulation to check the changes caused by three different sound sources, and natural music was recorded with acoustic, synthesizer, and neutral sound. For the stimulation method, in‐ear and bone conduction headphones were used to set the air conduction and bone conduction in Figure 2 as the stimulation location.

**TABLE 2:** The experimental procedure and stimulation method

Procedure	Time	Stimulation method	State
1	3	‐	Resting‐state, eyes open
2	3	‐	Resting‐state, eyes closed
3	3	In‐ear headphones	Native language song
4	3	In‐ear headphones	Non‐native language song
5	3	In‐ear headphones	Neutral music
6	3	Bone conduction headphones	Native language song
7	3	Bone conduction headphones	Non‐native language song
8	3	Bone conduction headphones	Neutral music

Reticular activating system activates the brain according to the inflow of sensory information. More precisely, with respect to actions and functions that spread throughout the brain, RAS regulates the cortical projection, which allows nerves to spread throughout the brain.^29^ Accordingly, Electrodes were placed at positions T7, F8, Cz, and P4 according to the EEG 10/10 system, and a reference electrode and a ground electrode were placed in the left and right ears.^28^ The electrode positions of T7, F8, Cz, and P4 overlap the EEG10/10 system and the EEG 10/20 system, and each subdivided brain region is observed. T is the temporal lobe, F is the frontal lobe, P is the parietal lobe, and finally, Cz is center between nasion and inion.^30^

### 2.3 EEG data analysis

Among the 3 min of data recorded in each procedure in Table 2, the EEG signal with less object movement and noise was divided to extract a total of 2 min of data. The extracted data were applied with a 1–40 Hz Butterworth filter and a 50 Hz notch filter with a quality factor of 30. EEG is a recording of the brain activity consisting of repeated waves with a constant cycle and waveform, and the amplitude may not be constant. For this reason, the power spectral density (PSD) of each frequency range was analyzed based on the period in Table 3.^31^ For analysis, the average value of the PSD was calculated using the Welch function of the SciPy signal module of Python. Welch's method computes a corrected periodogram for each segment and averages it to compute an estimate of the PSD. Finally, we analyzed data using paired t‐test to statistically verify the data change between the unstimulated steady state (procedure 1) and the stimulated state.

**TABLE 3:** EEG frequency analysis bands and descriptions

EEG waveform	Frequency band (Hz)	Situation in which the PSD is increased
Delta	0.5–4	Sleep and unconscious
Theta	4–8	Cognitive anxiety reduction and concentration improvement
Alpha	8–13	When the mind and body are at rest in the awakened state
Beta	13–30	A state of tension, anxiety, or concentration
Gamma	30–45	Extreme arousal and excitement

In order to confirm the possibility of cortical projection induction according to RAS stimulation, PSDs for each channel that change according to sound stimulation and stimulation method were analyzed. Thereafter, the PSD of a total of 20 waveforms was analyzed by subdividing the channel attached to each brain region into 5 signals according to the frequency range according to the EEG waveform in Table 3. These detailed waveforms can identify the active frequency range for each brain region, which can identify diseases and diseases, and detailed state changes of the subject. To confirm this, changes in mind wandering and focused attention and task‐related states^32^, ^33^ were observed through the theta/beta rate, which additionally indicates the functional and cognitive significance and alpha rate indicating the responsiveness to external stimulation.^34^

## 3 RESULTS

### 3.1 Power spectral density change according to the channel

**FIGURE 3:**
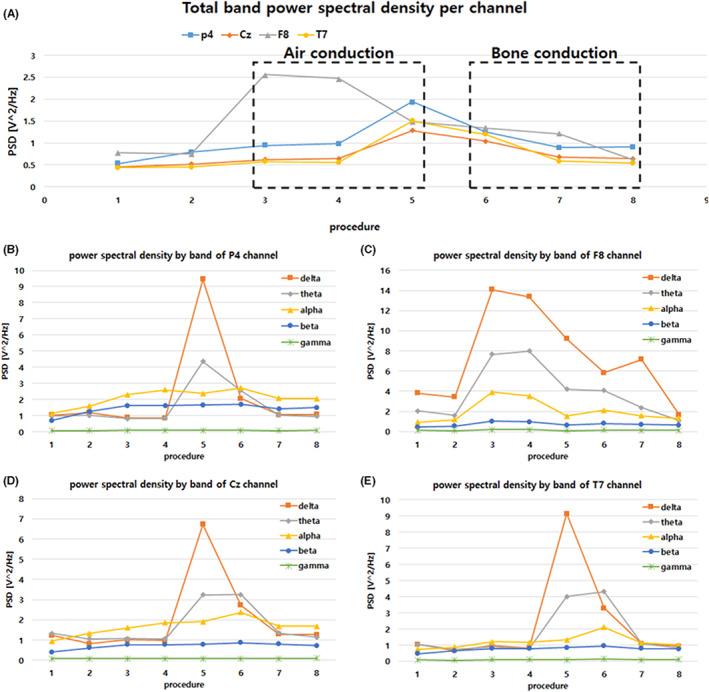
Total band power spectral density according to sound stimulus and method by channel. Figure shows the analysis of the electroencephalogram waveform for each electroencephalogram channel (P4: parietal lobe, Cz: central lobe, F8: frontal lobe, T7: Temporal lobe). The X axis means each procedure in Table 2. The duration of each procedure is 2 min. The Y‐axis represents the power spectral density. The higher the energy of the frequency in the band, the higher the Y value. Accordingly, it is possible to determine in what form the brain is being activated in each lobe. In addition, according to each procedure, we tried to observe changes in the brain according to the sound transmission method of air conduction and bone conduction and the type of sound stimulation. (A) shows EEG activity in the 0.5‐45 Hz band in the P4, Cz, F8, and T7 channel. (B), (C), (D) and (E) show the P4, F8, Cz and T7 channels, respectively, and the graphs show the delta, theta, alpha, beta, and gamma band activity in detail.

Figure 3A shows the total PSD for each channel that changed according to the experimental procedure. In procedure 1, the normal state, the PSD of the subjects was 0.55 ± 0.02 $V^{2}/\mathrm{Hz}$. However, according to the change of stimulation, PDS increased to 1.66 ± 0.11 $V^{2}/\mathrm{Hz}$ in procedure 5. As a result of closely observing this, the PSD in the delta band increased the most from 1.78 ± 1.86 to 8.70 ± 1.75 $V^{2}/\mathrm{Hz}$. Next, it was confirmed that the theta band increased from 1.34 ± 0.24 to 4.03 ± 0.29 $V^{2}/\mathrm{Hz}$. However, in Figure 3C, the PSD showed a sharp increase in procedure 3 in which the sound stimulation was started, and it showed a form that decreased with passage of time.

These results confirmed that even if the subjects were stimulated by the same sound, the change in the EEG was different depending on the method of air conduction and bone conduction. Among them, it was confirmed that the air conduction stimulation method and the sound stimulation with neutral music can lead to large changes in the delta and theta bands of EEG.

### 3.2 Power spectral density change according to the EEG band

**FIGURE 4:**
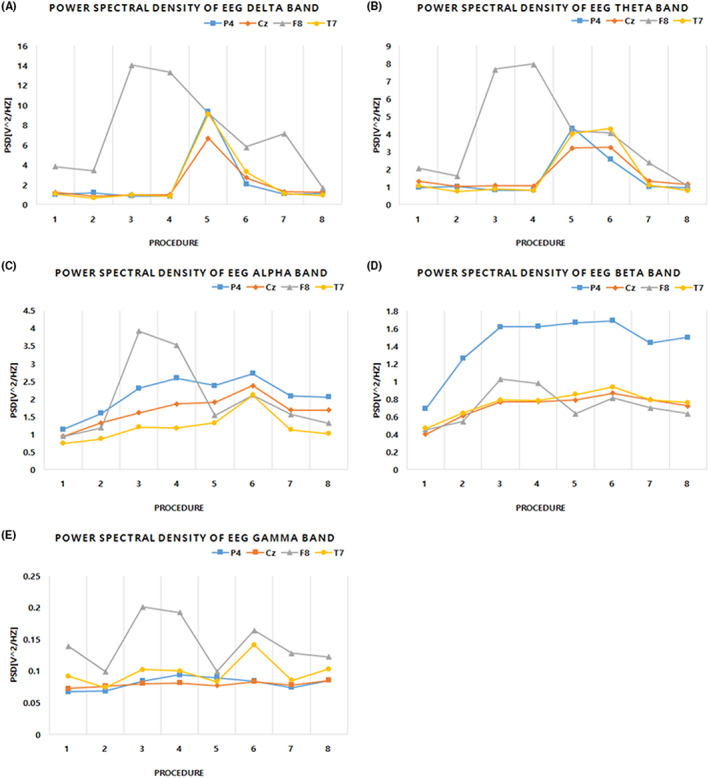
Power spectral density according to sound stimulus and method by band. The figure is intended to interpret the band‐specific changes of the channel region. The frequency bands of electroencephalogram have respective meanings as shown in Table 3. Detailed observation of each band can compare and analyze the effect of sound stimulation with existing EEG studies. The X‐axis and Y‐axis are the same as in Figure 3. (A), (B), (C), (D), and (E) represent the delta, theta, alpha, beta and gamma bands, respectively. The graph provides the change for each channel in each band (P4: parietal lobe, Cz: central lobe, F8: frontal lobe, T7: Temporal lobe).

As shown in Figures 3 and 4, channel F8 showed the highest response according to the frequency band. The PSD increased and then gradually decreased in procedure 3 of stimulation by air conduction and procedure 6 in which the stimulation started with bone conduction (Figures 4C–E). This point seemed to indicate that the frontal lobe was active at the moment when a sound stimulation or an external stimulation was performed. In addition, processes 3 and 6 were expected to have a correlation with language as the native music sound stimulation.

According to Figure 4A, it was confirmed that the effect could be different depending on the sound stimulation and stimulation method. In the delta band, procedure 5 and procedure 8 delivered the same sound stimulation through different methods, but only the air conduction stimulation used in procedure 5 induced significant EEG activity. This indicated that the auditory nerve stimulation that can change the delta band in bone conduction did not occur. Also, since the EEG changes for different sound stimulations were different in procedures 3, 4, and 5 using the same stimulation method, it appeared that a sound stimulus in a specific band can induce a change in EEG. However, while Figure 4A,B show a slight increase in procedure 6, we conclude that this increase is due to vibrational stimulation of bone conduction.

Finally, the PSD of channel P4, which was higher than that of other channels, was observed in Figure 4C,D. Because the parietal lobe, where P4 is located, is tasked with integrating and interpreting sensory input for gustatory, visual, tactile, olfactory, and auditory stimulation, it appears as an elevation of alpha and beta waveforms.

### 3.3 Observation of functional and cognitive changes in response to stimulation

**FIGURE 5:**
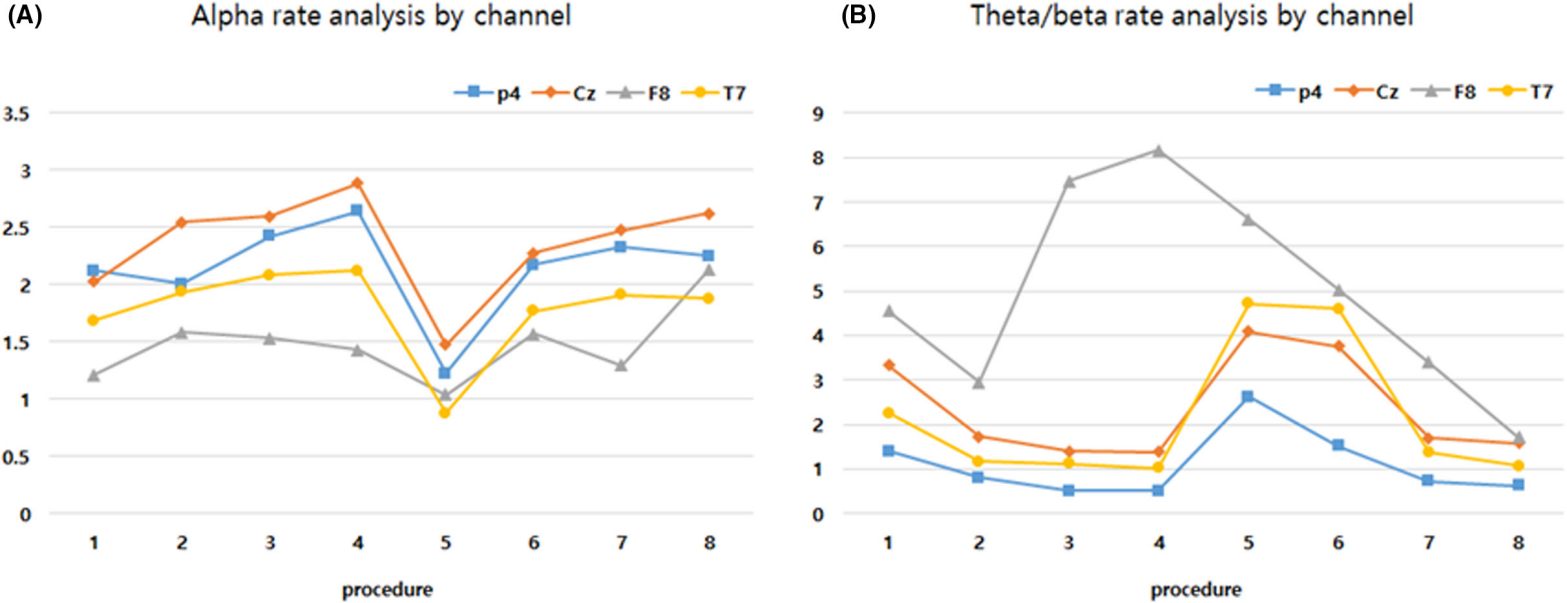
Changes in the final state of the subjects according to the sound stimulation. This figure is the content to analyze the final state of the subject from the changes shown in Figures 3 and 4. The X‐axis is the same as in Figures 3 and 4. The power in the alpha band in (A) confirms the subject's mental arousal state. The theta/beta power rate in (B) observes cognitive and functional changes. Through these values, it was attempted to infer the method of sound stimulation and the final state of the subject according to the stimulation sound

In general, the alpha band of EEG was activated when the subject was at rest in the state of mental arousal. However, as shown in Figure 5A, when neutral music was stimulated using the airway, the alpha ratio was significantly decreased from 1.76 ± 0.17 to 1.23 ± 0.03 (*p* < 0.05). In this case, it was similar to the mind wandering phenomenon called nonwork cognition, and this phenomenon represents cognition^34^, ^35^ oriented to the flow of internal thinking, and leads to immersion.^36^ Mind wandering was confirmed through theta/beta rate, which is one of the methods to more accurately confirm these cognitive and functional changes. Increased theta power and decreased beta power were observed during mind wandering, indicating a decrease in the executive control network and an increase in the default mode network (DMN).^32^ A change from 0.88 ± 0.05 to 0.46 ± 0.01 as shown in Figure 5B indicates that the subject entered a wandering state (*p* < 0.05).

## 4 DISCUSSION

Diseases of the brain and nervous system caused by aging, infection, and vascular problems pose a high economic and social burden. Neuropharmacological therapy and electrical stimulation are used as methods to treat the diseases, but safer treatment techniques are being studied because of the probability of various side effects and complications. Accordingly, in this study, the effect of a new treatment technique was assessed by analyzing the EEG signal of the nervous system that changes according to the sound stimulation and method using the concept of digital therapeutics, a new form of treatment for patients. Sound stimulation, such as music, has a good structure for expressing and recognizing specific emotions. In addition, because it has the ability to affect emotional, mental, and behavioral changes, it is being studied as a technology that supports the treatment of various behaviors and mental disorders.^37^

In Figure 3A, the increase in the PSD of channel F8 during sound stimulation and the increase in the PSD of channels P4, Cz, and T7 in procedure 5 were led by delta and theta bands. The delta band is associated with basic biological and homeostatic coordination. The activity of the RAS involved in cardiovascular system, respiratory and motor systems, and processing of the afferents generates delta and theta rhythms of EEG and induces cortical activity. In other words, it was confirmed that the stimulation of the auditory nerve using neutral music and air conduction method can induce activation of the RAS. This activity has the potential for synchronization with the vagus activity of the parasympathetic nervous system^38^, ^39^ and activation of the vagus nerve has clinical benefits that can be utilized for respiratory symptoms and respiratory therapy related to Coronavirus disease 2019 (COVID‐19).^24^

In Figure 4A,B, the auditory frequency range that can lead to delta and theta activation was determined to be 1 kHz. This means that the sound stimulation for inducing RAS activity should use a frequency band of less than 1 kHz. In Figures 3 and 4, in procedures 3, 4, and 5, sound stimulation was performed using the same air conduction stimulation method. However, the PSD of the delta and theta bands was increased only in procedure 5. The same sound stimulus used in procedure 5 did not make any changes when using the bone conduction method. Therefore, bone conduction is considered to be the reason behind why the frequency response of the cochlea is reduced at 1 kHz stimulation, and the skull and cranial soft tissues dissipate the stimulation energy in the low frequency region.^40^, ^41^, ^42^ Additionally, in procedure 6 in the theta band, the bone conduction method causes perception of mechanical vibrations other than auditory stimulation, and it is judged to be an increase caused by providing vibrotactile sensations in the nonauditory area.^43^, ^44^

The brain is made up of large cortical networks that are tightly coupled with high levels of cognition, and the DMN is active in the brain at rest, but it is deactivated when task performance begins. The DMN exhibits increased connectivity during mind wandering.^38^ The indicator of mind wandering is the theta/beta rate and it has a positive correlation. Mind wandering indicates a decrease in the sense of agency indicating mental and autonomy, and increased alpha and beta values. In Figures 4C,D, the alpha and beta values of the parietal region are increased, and it is known as a key region for the parietal‐occipital cortex connectivity analysis.^45^, ^46^ Thus, these results show that neutral music stimulation using air conduction can activate RAS stimulation and cortical connectivity, as shown in Figure 1.

Stress is a risk factor for a variety of diseases, ranging from metabolic and cardiovascular diseases to psychiatric diseases, and it can promote diseases. Short, controllable periods of stress are harmless to physical and mental health, but uncontrollable stress increases the susceptibility to related disorders and causes chronic distress.^47^ In severe cases, stress can lead to neurological and neuropsychiatric disorders, including Parkinson's disease, depression, attention deficit hyperactivity disorder, and mood disorders.^47^, ^48^ In this case, sedation is induced by a pharmacological method, and anesthesia is induced when sedation is gradually increased. The generally used anesthetic is propofol, and it is used medically due to its sedative and hypnotic effects. At this time, mild surgical‐level propofol anesthesia shows attenuation of the alpha and beta frequencies, as shown in Figure 5, and it significantly increases the power in the theta range.^49^ In other words, it was confirmed that the stimulation performed in this study has a value that can replace drugs by inducing changes in the nervous system similar to those induced by drugs for the sedative effect.

A limitation of this study is that the number of measured channels was small. However, the measurement method using the 10/10 system improved the accuracy of a specific area.^50^ A general EEG system follows the 10/20 system. This method was developed to maintain standardized test methods to collect, reproduce, and effectively analyze EEG, and it is based on the relationship between the cerebral cortex of the brain. Currently, more channels are used by the 10/10 system that can collect high‐resolution signals. These changes could improve the interpretation of the functional effects of stimuli in clinical settings.^51^ For this reason, it was judged that it would be possible to analyze the cortex with respect to changes in each part according to the sound stimulation and method.

In the other case, a clear frequency band analysis of the sound stimulus was insufficient. The source of the sound used consists of music in the native language, music in a foreign language, and neutral music. Finally, as a result of stimulation, it was determined that neutral music of less than 1 kHz using the air conduction method showed a clear effect. In other words, a definitive analysis of the music source used was not carried out. However, most of the sounds imitating nature, such as the sound of rain and water, have a band below 1 kHz. In addition, research on developing sound stimulation using individual biosignals as the reference values to quantitatively calculate the effect of treatment is also underway.^52^ Thus, it has great potential as a drug alternative treatment method that can provide quantitative values in the low frequency band.

## 5 CONCLUSION

This study analyzed the activation of the nervous system and cortex according to the sound stimulation and methods using the concept of digital therapy, which is a safer new treatment method than neuropharmacology and electrical stimulation. Accordingly, when stimulation with neutral music of less than 1 kHz was performed according to the air conduction method, the following results could be derived. The first is the parasympathetic activation effect. It shows the activity of the PAS and cortical connectivity, and among them, it shows the possibility of activation of the parasympathetic nervous system by stimulation of the vagus nerve. This parasympathetic nerve stimulation can be used for inspiratory symptoms and respiration therapy. The second is the sedative effect. The same EEG effect as that of a small amount of propofol anesthesia was confirmed. These results are expected to expand the concept of digital therapeutics, a new treatment method for preventing and treating diseases and disorders in patients. Finally, this study confirmed the possibility of RAS activity of sound stimulation by measuring EEG data of specific brain regions using 4 channels. Through future research, we plan to secure the association between new stimulation methods and neurofunctional brain connections^17^ to enable quantitative treatment.

## AUTHOR CONTRIBUTIONS

JHJ designed the data analysis method to secure the composition and clinical value of this study. DCK and JHW conducted thesis and data analysis focusing on drug substitution and brain activation potential based on the designed analysis method and digital therapeutic application method. Finally, SMK published a final review on the types and stimulation methods of acoustic stimulation, and all researchers shared their opinions to write a final review and conclusion.

## FUNDING INFORMATION

This study was carried out with the support of the Korea Medical Device Development Fund, funded by the government (Ministry of Science and Technology Information and Communication, Ministry of Trade, Industry and Energy, Ministry of Health and Welfare, Ministry of Food and Drug Safety) (Project identification number : RS‐2020‐KD000263, KMDF_PR_20200901_0263, NTIS_KMDK_RnD_1711138590)

## CONFLICT OF INTEREST

The authors declare no competing financial or nonfinancial interests. Also, the funders had no role in the design of the study in the collection, analyses, or interpretation of data in the writing of the manuscript or in the decision to publish the results.

## Data Availability

https://doi.org/10.13026/ps31‐fc50. The title of the raw data is Auditory evoked potential EEG‐biometric dataset. The analyzed data that support the findings of this study are available from the corresponding author upon reasonable request.
